# Supplementary figures and images for: Comprehensive Wet-Bench and Bioinformatics Workflow for Complex Microbiota Using Oxford Nanopore Technologies
Source: mSystems. 2021 Aug 24;6(4):e00750-21. doi: 10.1128/mSystems.00750-21 (PMC8407471; doi:10.1128/mSystems.00750-21)

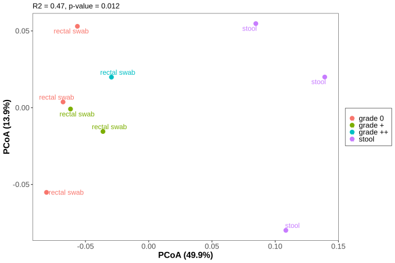

Supplement: FIG S1 [file msystems.00750-21-sf001.tif]

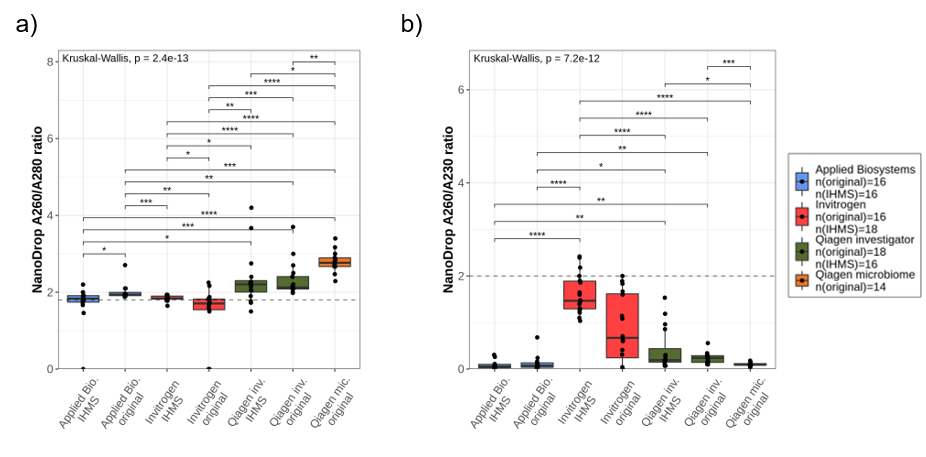

Supplement: FIG S2 [file msystems.00750-21-sf002.tif]

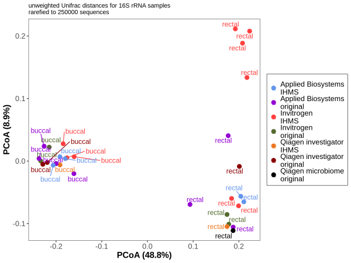

Supplement: FIG S3 [file msystems.00750-21-sf003.tif]

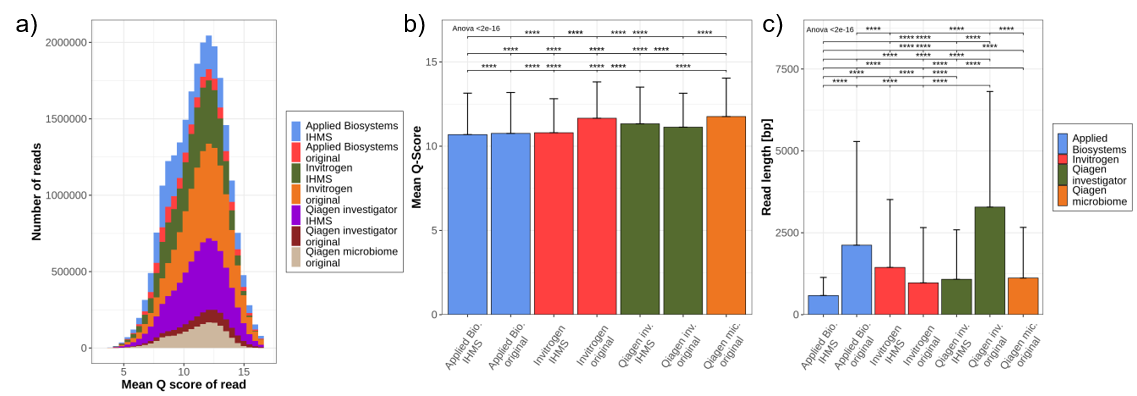

Supplement: FIG S4 [file msystems.00750-21-sf004.tif]

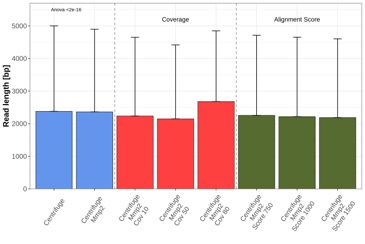

Supplement: FIG S5 [file msystems.00750-21-sf005.tif]
