# Supplementary material for: Comprehensive Wet-Bench and Bioinformatics Workflow for Complex Microbiota Using Oxford Nanopore Technologies
Source: mSystems. 2021 Aug 24;6(4):e00750-21. doi: 10.1128/mSystems.00750-21 (PMC8407471; doi:10.1128/mSystems.00750-21)
Supplement: TABLE S1 [file msystems.00750-21-st001.pdf]

Supplementary Table 1

Species	sample	stool_grade
Corynebacterium sp.	1	0
Corynebacterium jekeium	1	0
Akkermansia muciniphila	1	0
Corynebacterium sp.	2	0
Corynebacterium jekeium	2	0
Akkermansia muciniphila	2	0
Corynebacterium sp.	3	0
Corynebacterium jekeium	3	0
Akkermansia muciniphila	3	0
Corynebacterium sp.	4	+
Corynebacterium jekeium	4	+
Akkermansia muciniphila	4	+
Corynebacterium sp.	5	+
Corynebacterium jekeium	5	+
Akkermansia muciniphila	5	+
Corynebacterium sp.	6	+
Corynebacterium jekeium	6	+
Akkermansia muciniphila	6	+
Corynebacterium sp.	10	++
Corynebacterium jekeium	10	++
Akkermansia muciniphila	10	++
Corynebacterium sp.	11	++
Corynebacterium jekeium	11	++
Akkermansia muciniphila	11	++
Corynebacterium sp.	12	++
Corynebacterium jekeium	12	++
