# Supplementary material for: Comprehensive Wet-Bench and Bioinformatics Workflow for Complex Microbiota Using Oxford Nanopore Technologies
Source: mSystems. 2021 Aug 24;6(4):e00750-21. doi: 10.1128/mSystems.00750-21 (PMC8407471; doi:10.1128/mSystems.00750-21)
Supplement: TEXT S1 [file msystems.00750-21-s0001.docx]

# **Supplementary methods**

**Impact of eating and drinking on the buccal microbiome**.

The first swab was collected before breakfast, subsequent swabs followed 5, 30 and 240 min after eating. Afterwards 200 mL water were drunk and again buccal swabs were collected 5, 30 and 240 min after drinking, respectively. All swabs were frozen within three hours at -80 °C and DNA was isolated using the *PureLink™ Microbiome DNA Purification Kit* (Invitrogen) according to the IHMS protocol.

## **Classification**

For every sequence (readID) centrifuge could classify more than one taxonomic IDs (taxIDs). All sequences were excluded if the centrifuge score was below 150 or numbers of annotation exceeding 50 different taxIDs for a single readID. Sequences which passed these filters were validated with minimap2 by aligning sequences against their annotated reference FASTA files (55). These FASTA files and the corresponding taxIDs were extracted from the centrifuge indices using centrifuge-inspect. Cdbfasta (version 1.00) was applied to divide the complete FASTA file containing all sequences into files harbouring a sequence of a single organism (taxon). If more than one taxID was classified, the taxID with the highest normalized Smith Waterman Alignment Score (AS) and the best alignment coverages (Cov) was selected by a consecutive python script. Due to its dependence on the alignment length, the original Smith Waterman Score was divided by the alignment length (and multiplied by 1000 to get a better graduality). An alignment coverage of 50 % and a score of 1500 was set as default.

**Precision, recall, Area Under Precision Recall curve (AUPR)**

The precision is calculated by the number of true positive species divided by the total number of false positives in one sample (also known as positive predictive value). In contrast, recall is defined as the proportion of true positive species divided by species which are expected to be in the mock community (also known as sensitivity). Frequently, taxa were excluded below certain abundance thresholds in most microbiome analyses. Here, a single consideration of raw precision and recall will not be able to anticipate realistic classifier or library performance. To overcome this problem a precision recall curve was constructed by calculating these two scores for different abundance thresholds (ranging from 0-1.0). Afterwards the area under this curve (AUPR) was calculated as a single metric (57).

**Comparison of classifiers with simulated data**

Four classifiers were compared using four sets of simulated data: Kaiju, Kraken2, Metamaps,

and Metapont.

Kaiju

Kaiju v 1.7.4 is a metagenomic classifier which detects a maximum of exact matches (MEMs) on the protein-level using Burrows-Wheeler transform (62). NCBI BLAST nr + euk library was downloaded from the official website (last updated 16/05/2017) containing Bacteria, Archaea, Viruses, Fungi and microbial eukaryotes.

Kaiju command

kaiju -t {db.nodes} -f {db.fmi} -i {input} -o {output}-z {threads}

Kraken2

Kraken2 is a k-mer based classifier similar to Centrifuge and searches for 35 k-mers from the query sequence in a database that matches k-mers to the lowest common ancestor (LCA) (63). The library was created 20/06/2021 containing all genomes from the NCBI BLAST`s nt database.

Kraken2 command

kraken2 --db {database} --threads {} --output {} {fastq_file}

MetaMaps

MetaMaps is a classifier designed for long reads (ONT/ PacBio) sequenced with metagenomic approach (59). To date there is no comprehensive database available including all taxa from different kingdoms. To this end, the official preformed database containing 12000 genomes was applied.

MetaMaps command

unset MALLOC_ARENA_MAX

metamaps mapDirectly -t {threads} --all -r {DB.fa} -q {fastq-file} -o {classification_results} --maxmemory {memory}

metamaps classify -t 15 --mappings {classification_results} --DB {databases/miniSeq+H}

Metapont

Metapont uses Centrifuge for initial classification and Minimap2 as an alignment control. For simulated data a coverage of 50 and an alignment score of 1000 was applied. NCBI nt database was used as library.

## **International Human Microbiome Standards (IHMS) protocols**

**PureLink^TM^ Microbiome DNA Purification Kit**

1. Swab + 500µl of medium in 2ml bead-tubes, add 600µl S1 + 100µl S2
2. Vortex thoroughly
3. Incubate 15min at 95°C
4. Homogenize samples with bead-beating Precellys (10000rpm/6x 30s – 30s pause)
5. Incubate samples 5min on ice
6. Centrifuge for 5min with 16000g at 4°C
7. Transfer supernatant in new 2ml tubes
8. Add 300µl S1 + 50 µl S2 to bead-tubes and repeat Step 2-7
9. Transfer supernatant in new 2ml tubes
10. Add 260µl ammonium acetate 10M to the tube containing supernatant
11. Incubate on ice for 10min
12. Centrifuge for 10min with 16000g at 4°C
13. Transfer supernatant in 2ml tubes
14. Add 250µl S3
15. Vortex immediately to assure uniform mixture
16. Incubate 10min on ice
17. Centrifuge for 10min with 16000g at 4°C
18. Transfer supernatant in new 2ml tubes
19. Add 1ml isopropanol, vortex thoroughly and incubate on ice for at least 30min
20. **It is possible to stop protocol overnight, storing samples at 4°C**
21. Centrifuge for 15min with 16000g at 4°C
22. Discard supernatant
23. Wash pellet of DNA with 500µl ethanol (70%)
24. Dry samples with open lid for 15min
25. Alternatively centrifuge briefly and discard remaining liquid by pipetting
26. Resuspend pellet with 100µl TE (Tris-EDTA) (better resuspension after 10min incubation)
27. **It is possible to stop protocol overnight, storing samples at 4°**C
28. Add 2µl DNase free RNase (10mg/ml) and incubate at 37°C for 30min
29. Add 15µl proteinase K and 200µl S4, vortex and incubate 10min at 65°C
30. Add 200µl ethanol (100%) and vortex
31. Transfer total volume to column – centrifuge 1min with 14000g at 4°C
32. Wash again with 200µl of 100% Ethanol (incubation of 3min)
33. Discard elution and place column in a new tube
34. Add 500µl S5 into column – centrifuge 1min with 14000g at 4°C
35. Discard elution, place column in a new tube and repeat centrifugation of the column 1min with 14000g at 4°C
36. Place column in 1,5ml tube
37. Add 100µl S6
38. Incubate at room temperature for 5min
39. Centrifuge 1min with 14000g at 4°C

**MagMAX™ Microbiome Ultra Nucleic Acid Isolation Kit**

1. Swab + 500µl medium in 2ml bead tubes, add 800µl lysis buffer
2. Vortex thoroughly
3. Homogenize samples with bead-beating Precellys (10000rpm/6x 30s – 30s pause)
4. Incubate samples 5min on ice
5. Centrifuge for 5min with 16000g at 4°C
6. Transfer supernatant in new 2ml tubes
7. Add 400µl of Lysis buffer and repeat Step 2-7
8. Transfer supernatant in new 2ml tubes
9. Add 260µl ammonium acetate 10M to the tube containing supernatant
10. Incubate on ice for 10min
11. Centrifuge for 10min with 16000g at 4°C
12. Transfer supernatant in 2ml tubes
13. Add 1ml isopropanol, vortex thoroughly and incubate on ice for at least 30min
14. **It is possible to stop protocol overnight, storing samples at 4°C**
15. Centrifuge for 15min with 16000g at 4°C
16. Discard supernatant
17. Wash pellet of DNA with 500µl ethanol (70%)
18. Dry samples with open lid for 15min
19. Alternatively centrifuge briefly and discard remaining liquid by pipetting
20. Resuspend pellet with 100µl TE (Tris-EDTA) (better resuspension after 10min incubation)
21. **It is possible to stop protocol overnight, storing samples at 4°**C
22. Add 2µl DNase free RNase (10mg/ml) and incubate at 37°C for 30min
23. Add 40 μL of Proteinase K to each sample and shake samples at 900 rpm for 5 minutes.
24. Place samples in an incubator at 65°C for 20 minutes.
25. Invert Binding Bead Mix to mix, then add 520 μL to each sample in the Sample Plate.
26. Shake the mix at 900 rpm for 5 minutes.
27. Place the tubes on the magnetic stand for at least 5 minutes, or until all the beads have collected.
28. Keeping the samples on the magnet, carefully remove the cover, then discard the supernatant from each well. IMPORTANT: Avoid disturbing the beads
29. Remove the tubes from the magnetic stand, then add 1 mL of Wash Buffer to each sample.
30. Shake at 800 rpm for 30 seconds.
31. Place the tubes back on the magnetic stand for 3 minutes, or until all the beads have collected.
32. Keeping the samples on the magnet, carefully remove the cover, then discard the supernatant from each well. IMPORTANT! Avoid disturbing the beads.
33. Repeat step 37 to step 40 using 1 mL of Wash Buffer.
34. Repeat step 37 to step 40 using 1 mL of 80% Ethanol.
35. Repeat step 37 to step 40 using 1 mL of 80% Ethanol.
36. Dry the beads by shaking the tubes (uncovered) at 800 rpm for 2 minutes.
37. Add 50 μL of Elution Solution to each sample
38. Place the samples in an incubator at 75°C for 5 minutes.
39. Place tubes on a shaker at 800 rpm for 5 minutes.
40. Place tubes on the magnetic stand for 3 minutes or until all beads are collected against the magnets.
41. Transfer the eluates to a fresh 1,5 LowBind Tube

**QIAamp DNA Investigator Kit**

1. Swab + 500µl of medium in 2ml bead-tubes (e.g. from Invitrogen Kit), add 400µl ATL buffer + 20µl Proteinase K
2. Vortex thoroughly
3. Incubate 60 min at 56°C with 900rpm
4. Add 400µl AL buffer, vortex thoroughly
5. Incubate 15min at 95°C with 900rpm
6. Homogenize samples with bead-beating Precellys (10000rpm/6x 30s – 30s pause)
7. Incubate samples 5min on ice
8. Centrifuge for 5min with 16000g at 4°C
9. Transfer supernatant in new 2ml tubes
10. Add 300µl AL buffer and repeat Step 5-8
11. Transfer supernatant in new 2ml tubes
12. Add 260µl ammonium acetate 10M to the tube containing supernatant
13. Incubate on ice for 10min
14. Centrifuge for 10min with 16000g at 4°C
15. Transfer supernatant in 2ml tubes
16. Add 1ml isopropanol, vortex thoroughly and incubate on ice for at least 30min
17. **It is possible to stop protocol overnight, storing samples at 4°C**
18. Centrifuge for 15min with 16000g at 4°C
19. Discard supernatant
20. Wash pellet of DNA with 500µl ethanol (70%)
21. Dry samples with open lid for 15min
22. Alternatively centrifuge briefly and discard remaining liquid by pipetting
23. Resuspend pellet with 100µl TE (Tris-EDTA) (better resuspension after 10min incubation)
24. **It is possible to stop protocol overnight, storing samples at 4°**C
25. Add 200µl ethanol (96-100%) close the lid, and mix by pulse-vortexing for 15 s
26. Briefly centrifuge the 2 ml tube to remove drops from the inside of the lid.
27. Transfer the entire lysate from step 9 to the QIAamp MinElute column (in a 2 ml collection tube)
28. Add 500 μl Buffer AW1 and centrifuge at 6000 x g (8000rpm) for 1 min, place the QIAamp MinElute column in a clean 2 ml collection tube
29. Add 700 μl ethanom (96-100%) and centrifuge at 6000 x g (8000rpm) for 1 min, place the QIAamp MinElute column in a clean 2 ml collection tube
30. Centrifuge at full speed (20,000 x g; 14,000 rpm) for 3 min
31. Place the QIAamp MinElute column in a clean 1.5 ml microcentrifuge tube and incubate at room temperature (15–25°C) for 10 min or at 56°C for 3 min with open lid
32. Add 50 μl Buffer ATE or distilled water to the center of the membrane, incubate for 5min at room temperature
33. Centrifuge at full speed (20,000 x g; 14,000 rpm) for 1 min
