# Supplementary material for: Comprehensive Wet-Bench and Bioinformatics Workflow for Complex Microbiota Using Oxford Nanopore Technologies
Source: mSystems. 2021 Aug 24;6(4):e00750-21. doi: 10.1128/mSystems.00750-21 (PMC8407471; doi:10.1128/mSystems.00750-21)
Supplement: TABLE S2 [file msystems.00750-21-st002.pdf]

Supplementary Table 2

Species	phv	p	ncbi complete	nt database
Lactobacillus fermentum	x	x	x	x
Escherichia coli	x	x	x	x
Bacteroides fragilis	x	x	x	x
Fusobacterium nucleatum	x	x	x	x
Roseburia hominis	x	x	x	x
Bifidobacterium adolescentis	x	x	x	x
Akkermansia muciniphila	x	x	x	x
Clostridioides difficile	x	x	x	x
Salmonella enterica	x	x	x	x
Enterococcus faecalis	x	x	x	x
Clostridium perfringens	x	x	x	x
Faecalibacterium prausnitzii	-	x	x	x
Methanobrevibacter smithii	-	x	x	x
Saccharomyces cerevisiae	-	-	x	x
Candida albicans	-	-	-	x
Veillonella rogosae	-	-	-	x
Prevotella corporis	-	-	-	x
