# Supplementary material for: Comprehensive Wet-Bench and Bioinformatics Workflow for Complex Microbiota Using Oxford Nanopore Technologies
Source: mSystems. 2021 Aug 24;6(4):e00750-21. doi: 10.1128/mSystems.00750-21 (PMC8407471; doi:10.1128/mSystems.00750-21)
Supplement: TABLE S3 [file msystems.00750-21-st003.pdf]

Supplementary Table 3

10 taxa		tumor		gut	
Species	Abundances	Species	Abundances	Species	Abundances
Treponema pallidum	0,1	Homo sapiens	0,999	Faecalibacterium prausnitzii	0,14
Streptococcus pneumoniae	0,1	Bacillus clausii	0,000125	Veillonella dispar	0,14
Staphylococcus aureus	0,1	Citrobacter freundii	0,000125	Roseburia hominis	0,14
Serratia marcescens	0,1	Enterobacter asburiae	0,000125	Bacteroides fragilis	0,14
Escherichia coli	0,1	Enterobacter cloacae	0,000125	Escherichia coli	0,14
Legionella pneumophila	0,1	Fusobacterium nucleatum	0,000125	Prevotella intermedia	0,06
Klebsiella pneumoniae	0,1	Klebsiella pneumoniae	0,000125	Bifidobacterium adolescentis	0,06
Bacillus cereus	0,1	Lactobacillus iners	0,000125	Fusobacterium nucleatum	0,06
Candida albicans	0,1	Staphylococcus aureus	0,000125	Lactobacillus fermentum	0,06
Aspergillus fumigatus	0,1			Clostridioides difficile	0,015
				Akkermansia muciniphila	0,015
				Candida albicans	0,015
				Saccharomyces cerevisiae	0,015
				Methanobrevibacter smithii	0,001
				Salmonella enterica	0,0001
				Enterococcus faecalis	0,00001
				Clostridium perfringens	0,000001

Metamaps					
Species	Abundances	Species	Abundances	Species	Abundances
Alkalilimnicola ehrlichii	0,0130	Escherichia coli	0,0130	Pseudomonas stutzeri	0,0130
Alkaliphilus metalliredigens	0,0130	Francisella tularensis	0,0130	Psychrobacter cryohalolentis	0,0130
Bacillus anthracis	0,0130	Geobacter sulfurreducens	0,0130	Psychromonas ingrahamii	0,0130
Bacillus cereus	0,0130	Haemophilus influenzae	0,0130	Cupriavidus necator	0,0130
Bacillus clausii	0,0130	Histophilus somni	0,0130	Rhodococcus jostii	0,0130
Bacillus halodurans	0,0130	Haloquadratum walsbyi	0,0130	Rhodopseudomonas palustris	0,0130
Bacillus subtilis	0,0130	Hyphomonas neptunium	0,0130	Hungateiclostridium thermocellum	0,0130
Bacillus thuringiensis	0,0130	Idiomarina loihiensis	0,0130	Salmonella enterica	0,0130
Bacteroides thetaiotaomicron	0,0130	Ignicoccus hospitalis	0,0130	Shewanella sp.	0,0130
Bordetella bronchiseptica	0,0130	Lactobacillus delbrueckii	0,0130	Sinorhizobium meliloti	0,0130
Borreliella bavariensis	0,0130	Lactobacillus salivarius	0,0130	Sodalis glossinidius	0,0130
Buchnera aphidicola	0,0130	Lactococcus lactis	0,0130	Staphylococcus aureus	0,0130
Burkholderia pseudomallei	0,0130	Leptospira borgpetersenii	0,0130	Streptococcus agalactiae	0,0130
Campylobacter concisus	0,0130	Listeria welshimeri	0,0130	Streptococcus pneumoniae	0,0130
Chlamydia trachomatis	0,0130	Methanococcus maripaludis	0,0130	Streptococcus pyogenes	0,0130
Chlamydia caviae	0,0130	Mycobacterium avium	0,0130	Synechococcus elongatus	0,0130
Chlamydia pneumoniae	0,0130	Mycobacterium tuberculosis	0,0130	Syntrophomonas wolfei	0,0130
Pelodictyon luteolum	0,0130	Mycobacterium marinum	0,0130	Thermosipho melanesiensis	0,0130
Chromobacterium violaceum	0,0130	Mycobacterium tuberculosis	0,0130	Thermus thermophilus	0,0130
Clostridium beijerinckii	0,0130	Neisseria gonorrhoeae	0,0130	Thiobacillus denitrificans	0,0130
Clostridium botulinum	0,0130	Neisseria meningitidis	0,0130	Treponema denticola	0,0130
Clostridium novyi	0,0130	Nostoc punctiforme	0,0130	Ureaplasma parvum	0,0130
Corynebacterium efficiens	0,0130	Ochrobactrum anthropi	0,0130	Wigglesworthia glossinidia	0,0130
Corynebacterium urealyticum	0,0130	Paraburkholderia phymatum	0,0130	Yersinia pestis	0,0130
Coxiella burnetii	0,0130	Prochlorococcus marinus	0,0130		
Cytophaga hutchinsonii	0,0130	Pseudomonas putida	0,0130		
